# Investigating the Impact of Varied Dietary Protein Levels on *Litopenaeus vannamei*: An Exploration of the Intestinal Microbiota and Transcriptome Responses

**DOI:** 10.3390/ani14030372

**Published:** 2024-01-24

**Authors:** Gongyu Li, Hang Yuan, Zhibin Fu, Xinghui Luo, Zhihao Xue, Shuang Zhang

**Affiliations:** 1 College of Fisheries, Guangdong Ocean University, Zhanjiang 524088, China; ligongyu200007@163.com (G.L.);; 2 Key Laboratory of Aquatic, Livestock and Poultry Feed Science and Technology in South China, Ministry of Agriculture, Zhanjiang 524088, China; 3 Aquatic Animals Precision Nutrition and High Efficiency Feed Engineering Research Center of Guangdong Province, Zhanjiang 524088, China; 4 Guangdong Provincial Key Laboratory of Aquatic Animal Disease Control and Healthy Culture, Zhanjiang 524088, China

**Keywords:** *Litopenaeus vannamei*, intestinal microbiota, transcriptome sequencing, growth, immunity

## Abstract

**Simple Summary:**

Five feed protein levels (32%, 36%, 40%, 44%, and 48%) were set up using fishmeal as the sole protein source to study the effects of different feed protein levels on the intestinal microbiota and transcriptome responses of *L. vannamei*. It was found that the optimal feed protein level for shrimp *L. vannamei* is between 40% and 44%. Excessively high or low feed protein levels reduce antioxidant levels and digestive enzyme activity and promote pathogen colonization. The transcriptional regulation of inappropriate protein levels responds to the body by reducing factors in various metabolic pathways. The weakened genes impede metabolic processes and compromise immunological function, increasing the organism’s vulnerability to diseases. This can impair the immune systems of shrimp and cause stunted growth.

**Abstract:**

This study explored the effects of dietary protein levels on *Litopenaeus vannamei* with its intestinal microbiota and transcriptome responses. Previous studies on the effects of dietary protein levels on *L. vannamei* have focused on growth performance, antioxidant indices, and digestive enzyme activity, but few studies have been conducted at the microbiological and molecular levels. In this study, five isolipid experimental diets with protein levels of 32% (P32), 36% (P36), 40% (P40), 44% (P44), and 48% (P48) were used in an *L. vannamei* (0.63 ± 0.02 g) feeding trial for 56 days. At the end of the feeding trial, the growth performance, immunity, intestinal health, and transcriptional responses of *L. vannamei* were determined. This study demonstrated that higher protein levels (P44) led to superior weight gain and growth rates for *L. vannamei*, with lower feed conversion ratios (FCR) observed in the P48 and P44 groups compared to the P32 and P36 groups (*p* ≤ 0.05). The P44 and P48 groups also showed a notably higher protein efficiency ratio (PER) compared to others (*p* ≤ 0.05), and there was no significant difference between them. Upon *Vibrio parahaemolyticus* infection, the P48 group exhibited a significantly lower survival rate (SR) within 48 h, while during 72 h of white spot syndrome virus (WSSV) infection, the P44 group had a notably higher survival rate than the P32 group (*p* ≤ 0.05). Digestive enzyme activity and antioxidant levels in *L. vannamei* initially increased and then decreased as protein levels increased, usually peaking in the P40 or P44 groups. Lower dietary protein levels significantly reduced the relative abundance of beneficial bacteria and increased the relative abundance of pathogenic bacteria in the intestines of *L. vannamei*. Transcriptome sequencing analysis revealed that most differentially expressed genes (DEGs) were up-regulated and then down-regulated as dietary protein levels increased. Furthermore, KEGG pathway enrichment analysis indicated that several immune and metabolic pathways, including metabolic pathways, glutathione metabolism, cytochrome P450, and lysosome and pancreatic secretion, were significantly enriched. In summary, the optimal feed protein level for *L. vannamei* shrimp was 40–44%. Inappropriate feed protein levels reduced antioxidant levels and digestive enzyme activity and promoted pathogen settlement, deceasing factors in various metabolic pathways that respond to microorganisms through transcriptional regulation. This could lead to stunted growth in *L. vannamei* and compromise their immune function.

## 1. Introduction

The setting of dietary protein levels is critical and directly affects the growth, health, and overall production performance of aquatic animals, which, in turn, determines whether or not aquaculture practices are profitable [1,2]. A balanced protein diet ensures efficient energy utilization and facilitates essential biochemical reactions. Conversely, insufficient protein levels may result in stunted growth, delayed molting, and reduced health [3]. The optimal protein content in feed can vary based on the species, life stage, and environmental conditions [4]. For instance, juvenile shrimp generally require greater protein intakes than adult shrimp [5]. Appropriate protein levels in feed maximize its benefits, while inappropriate protein levels may cause indigestion. Additionally, low-quality proteins may result in nutrient waste and deficiencies, while excessive protein contents in feed can also deteriorate the quality of water due to the breakdown of surplus proteins, negatively impacting the overall health of the aquatic environment [6]. However, because proteins often represent a substantial expense in aquafeed production, it is crucial to balance their levels with other nutrients for cost-effectiveness [7]. Martinez-Cordova et al. conducted a cost analysis, revealing that employing a high protein feed during breeding was not the most optimal feeding strategy for *Litopenaeus vannamei* [8]. This demonstrated that feeding high protein levels is not optimal under certain circumstances.

*L. vannamei* has a significant economic importance in global aquaculture. Its prolific breeding yield and substantial economic returns have prompted extensive research into understanding and enhancing its production efficiency [9,10]. Variations in dietary protein levels can have multifaceted effects on *L. vannamei*. Xu et al. demonstrated that alterations in protein content impacted the plasma and hepatopancreas total antioxidant capacity as well as decreased the glutathione/oxidized glutathione ratios in plasma, potentially disrupting both the antioxidant capacity and immune responses of the shrimp [11]. In a study examining the influence of protein levels on trypsin activity, it was observed that changes in dietary protein content coupled with induced stress-affected trypsin activity in juvenile *L. vannamei* [12]. The dynamic interplay between the dietary protein content and environmental factors has also been investigated from various angles. Jang et al. investigated the impact of dietary protein levels on whole-body proximate amino acid composition and key waste nitrogen excretion parameters, including ammonia, nitrite, and nitrate nitrogen. The emission of these metabolic by-products into the environment induced environmental stress [13]. In another study, Jatobá et al. reported that utilizing a feed with 30.3% crude protein in a biological flocculation system reduced the feed costs for shrimp, offering potential savings in raw feed materials [14]. Limited studies have examined the impact of protein levels on *L. vannamei* at the molecular level. The effects of supplementing juvenile shrimp diets with 484.6 g/kg of protein were examined by Xie et al. in 2020. They observed higher mRNA expression levels of the target of rapamycin protein (TOR) and eukaryotic translation initiation factor 4e-binding protein (4EBP), along with significant alterations in the expression of two pivotal translation initiation factors, eukaryotic translation initiation factor 4E (eif4e) and eukaryotic translation initiation factor 3k (eif3k). Additionally, three endoplasmic reticulum (ER) stress-associated genes (eif2α, xbp1, and ayf4) were found to be influenced by changes in protein levels [5].

However, while previous studies on the effects of dietary protein levels on *L. vannamei* focused on macro-indicators such as growth performance and digestive enzyme activity, there have been few studies on these effects at the microbial and molecular levels. With the increasing sophistication of sequencing technologies, microbial and transcriptome sequencing tools have been widely utilized in many studies for various purposes, including examining the diversity and composition of the intestinal microbiota in response to varying proteins in the diet [15,16]; identifying the specific genes and pathways that are differentially expressed in response to differential feeding, shedding light on the molecular mechanisms underlying growth or physiological changes [17]; and, integrating data from both microbial sequencing and transcriptomics to gain a holistic view of the relationship between feed, intestinal microbiota, and host gene expression [18].

The association between dietary nutrition and the intestinal microbial community’s structural composition of the host has garnered increasing research attention. The intestinal tract serves as a site for nutrient absorption and accommodates a significant microbial population. The composition of the microbial communities plays a pivotal role in shaping host health and preserving homeostasis [19,20]. Furthermore, the diversity of the intestinal microbiota facilitates the presence of latent pathogens while concurrently hosting beneficial bacteria whose metabolites can be absorbed, thereby influencing the internal environment of the host [21]. Transcriptomics analysis is a potent diagnostic tool that is frequently employed in shrimp nutrition and immunity research, offering a comprehensive understanding of gene expression and its implications [22]. Zhen et al. explored the alterations in the whole transcriptome of *L. vannamei* induced by chronic imidacloprid exposure, providing valuable insights [23]. In another study, the authors identified that differentially expressed genes (DEGs) related to detoxification were associated with the intestinal microbiota, which sheds light on the competitive dynamics among specific bacteria [24]. Jia et al. utilized transcriptomic analysis to probe into the adverse effects of low fishmeal on the metabolic response of *L. vannamei* [25]. Thus, this technology empowers researchers to dissect the molecular mechanisms underlying the response of *L. vannamei* to endogenous and exogenous environmental factors.

This study aimed to shed light on the impact of varying the protein feed level, using fishmeal as the sole protein source, on the structure of the intestinal microbiota and the transcriptome profile of *L. vannamei*. This provided a deeper understanding of the protein requirements of species, furnishing valuable insights to further advance feed research and establish a theoretical framework for the optimization of feed usage.

## 2. Materials and Methods

### 2.1. Experimental Diets

The feed formulations utilized are detailed in Table 1, where fishmeal served as the exclusive protein source in five feeds denoted as P32, P36, P40, P44, and P48, representing protein levels of 32%, 36%, 40%, 44%, and 48%, respectively. Raw materials underwent crushing and screening through an 80-mesh screen. Subsequently, they were meticulously mixed using a step-by-step expanding method and homogenized in a V-type mixer (JS-14S, Zhejiang Chint Electrics Co., Ltd., Yueqing, China). Additional ingredients like fish oil, corn oil, and soybean lecithin were incorporated and thoroughly blended before extrusion through a twin-screw extruder (M-256, South China University of Technology, Guangzhou, China). The resulting feed particles were cooked for 20 min in a drying box (Shanghai Yiheng Scientific Instrument Co., Ltd., Shanghai, China, DHG-9240A) at 75 °C and stored in a refrigerator at 20 °C after being air-dried at natural temperatures.

### 2.2. Shrimp and Feeding Management

*L. vannamei* larvae were sourced from Zhanjiang Yuehai Aquatic Fry Co., Ltd. (Zhanjiang, China) consisting of 600 individuals with an initial weight averaging 0.63 ± 0.02 g. These were randomly allocated into five equal groups and fed with P32, P36, P40, P44, and P48 diets. Each subgroup comprised three biological replicates, with 40 individuals placed in separate 300-L fiberglass tanks. The stocking density of the experimental shrimp was about 133/m^3^, using indoor recycled water systems. Feeding occurred four times daily at 7:00, 11:00, 17:00, and 21:00. Initially, *L. vannamei* shrimp were fed several feeds equivalent to 10% of their body weight. Feeding was continued until satiety was reached, and the amount fed each day after that was determined using the amount consumed the day before. Implementing a daily water change of 50% while closely monitoring and regulating the temperature (by measuring the water temperature prior to each change and adjusting the percentage of water change accordingly, maintaining it within the range of 27–30 °C), ammonia nitrogen (≤0.05 mg/L), salinity (27–30 ppt), dissolved oxygen (>6.0 mg/L), and pH (7.7–8.0) were all carried out to guarantee ideal circumstances. Water quality was assessed after routine alterations. AR8210, PH828, and AR8212 from Dongguan SMART SENSOR Instrument Co., Ltd. (Dongguan, China), and the RB-103C Portable Ammonia Nitrogen Rapid Measuring Instrument from Guangzhou Ruibin Technology Co., Ltd. (Guangzhou, China), were used to measure the levels of dissolved oxygen salinity, ph and ammonia nitrogen.

### 2.3. Growth Performance Analysis

At the end of the feeding trial, the shrimp were counted and weighed to determine their growth performance indicators, including their survival rate (SR), final body weight (FW), weight gain rate (WGR), specific growth rate (SGR), feed conversion ratio (FCR), and protein efficiency ratio (PER). They were computed based on these parameters.

$$SR\;(\%)=\frac{Final\;shrimp\;number}{Initial\;shrimp\;number}\times 100$$

$$WGR\;(\%)=\frac{\left(Final\;body\;weight-Initial\;body\;weight\right)}{Initial\;body\;weight}$$

$$SGR\;(\%\;d^{-1})=\frac{\left[Ln\;(Final\;body\;weight)-Ln\;(Initial\;body\;weight)\right]}{Days}$$

$$FCR=\frac{Feed\;intake}{\left(Final\;body\;weight-Initial\;body\;weight\right)}$$

$$PER\;(\%)=\frac{\left(Final\;body\;weight-Initial\;total\;weight\right)}{Protein\;intake}\times 100$$

### 2.4. Sample Collection

The shrimp were sampled after 8 weeks of culture and were fasted for 24 h before sampling. Six randomly selected shrimp from each tank were sampled. Hemolymph was extracted from the pericardial cavity using a 1-mL sterile syringe, while the muscle, hepatopancreas, and intestinal tissues were carefully separated. Tissues from three shrimp were combined into a single sample and placed in separate 1.5-mL centrifuge tubes. One tube containing the hemolymph was centrifuged at 3000× *g* for 5 min at 4 °C, precipitating it. The supernatant was removed, and 1 mL of RNAlater™ (Thermo Fisher, Waltham, MA, USA) was added to resuspend the hemolymph, preserving it at −80 °C for subsequent transcriptome sequencing and gene expression analyses. The other tube containing tissue samples was promptly frozen in liquid nitrogen and stored at −80 °C for subsequent enzyme activity assays, body composition analysis, and intestinal microbiome sequencing.

### 2.5. Challenge Tests

White spot syndrome virus (WSSV) isolates and *Vibrio parahaemolyticus* strains were provided by the Crustacean Laboratory of Guangdong Ocean University (Zhanjiang, China). Preparations of WSSV and *V. parahaemolyticus* were carried out, and the challenge concentrations of them were determined according to our previous studies [26,27]. The WSSV isolates and *V. parahaemolyticus* strains were provided by the Crustacean Laboratory of Guangdong Ocean University (Zhanjiang, China). After the completion of sample collection, 40 shrimp from a group were transferred to a 250-L experimental drum for one week to reach a steady state. After feeding was stopped for 24 h, they were then divided into two groups for the challenge tests with WSSV and *V. parahaemolyticus*. For the challenge test by WSSV, a total of 20 shrimp were chosen at random from each group and subjected to an exposure of WSSV at a concentration of 1 × 10^5^ copies/g shrimp. The shrimp mortality rate was closely observed and documented every 4 h for 120 h. For the challenge test by *V. parahaemolyticus*, the remaining 20 shrimp were chosen at random from each group and subjected to an exposure of *V. parahaemolyticus* at a concentration of 1 × 10^7^ CFU/g shrimp. The cumulative mortality rate was recorded every 4 h for 72 h. The water quality conditions during the respite and testing periods remained consistent with those of the feeding trial experiment. No feed was provided during the experiments. The differences among groups were assessed using the Mantel–Cox (log-rank χ^2^ test) method in GraphPad Prism 9.0.

### 2.6. Non-Specific Immune Indices

The stored hepatopancreas samples maintained at −80 °C were thawed, weighed, and ground, and then diluted with 0.90% saline at 4 °C in a ratio of 1:9. The resulting mixtures underwent centrifugation at 4000 rpm for 15 min at 4 °C, followed by the removal of the supernatant. Subsequently, the activities of lysozyme (LZM), superoxide dismutase (SOD), catalase (CAT), and phenoloxidase (PO), and the content of malondialdehyde (MDA) were determined with the help of a Multiskan Spectrum Microplate Spectrophotometer (Thermo, MultiskanGO1510) using the enzyme activity assay kits A050-1-1, A001-3-2, A007-2-1, H247, and A003-1-2, respectively. The enzyme activity assay kits were all brought from the Nanjing Jiancheng Institute of Bioengineering (Nanjing, China), and the experimental schemes were strictly following the manufacturer’s instructions.

### 2.7. Digestive Enzyme Activity

The intestinal samples stored in a refrigerator at a −80 °C were thawed and weighed. A total of 0.5 g of intestine were weighed, homogenized, and diluted with 0.9% saline at a ratio of 1:9 at 4 °C. The sample was then centrifuged at 4 °C (4000 rpm) for 15 min, and the supernatant was extracted to determine the amylase (AMS), trypsin, and lipase activities of the intestines using the enzyme activity assay kits C016-1-1, A080-2-2, and A054-2-1, respectively, with the help of a Multiskan Spectrum Microplate Spectrophotometer (Thermo, MultiskanGO1510). The enzyme activity determination kits were all obtained from the Nanjing Jiancheng Institute of Bioengineering, and the experimental scheme was strictly following the manufacturer’s instructions.

### 2.8. Intestinal Microbial Analysis

The genomic DNA extraction of microorganisms from the intestinal samples followed the manufacturer’s protocol using HiPure Soil DNA Kits (Magen, Guangzhou, China). The amplification of the V3–V4 region of the 16S rDNA gene was achieved using the primers 341F (CCTACGGGNGGCWGCAG) and 806R (GGACTACHVGGGTATCTAAT). The PCR program involved an initial denaturation step at 95 °C for 5 min, followed by 30 cycles at 95 °C for 1 min, 60 °C for 1 min, 72 °C for 1 min, and a final extension at 72 °C for 7 min. PCR reactions were conducted in triplicate 50-μL mixtures consisting of 10 μL of 5 × Q5@ Reaction Buffer, 10 μL of 5 × Q5@ High GC Enhancer, 1.5 μL of 2.5-mM dNTPs, 1.5 μL of each primer (10 μM), 0.2 μL of Q5@ High-Fidelity DNA Polymerase, and 50 ng of template DNA. PCR reagents were sourced from New England Biolabs, USA. Subsequently, amplicons were purified using the AxyPrep DNA gel extraction kit (Axygen Biosciences, Union City, CA, USA), pooled in equimolar concentrations, and sequenced via a Hiseq2500 PE250 machine (Illumina, San Diego, CA, USA) at Guangzhou Genedenovo Biotechnology Co., Ltd. (Guangzhou, China) The raw sequencing data were deposited in NCBI GenBank (http://www.ncbi.nlm.nih.gov/genbank/, accessed on 12 December 2023).

FASTP [15] was employed to filter the raw reads further and eliminate noise sequences under specific conditions to obtain high-quality, clean reads. This process generated high-quality clean tags. These clean tags underwent clustering into operational taxonomic units (OTUs) of ≥97% similarity using the UPARSE pipeline [28]. The representative OTU sequences were classified using the RDP classifier based on the SILVA database [29,30], employing a confidence threshold of 0.8. Alpha diversity indexes, including OTUs, Chao1, ace, Shannon, Simpson, and Goods coverage, were computed using QIIME [31,32]. The sequencing information had the accession number PRJNA1061753 and could be found in the NCBI GenBank database.

### 2.9. Transcriptome Analysis

Total RNA extraction from the samples followed the Trizol method. As over 90% of RNA in typical species comprises rRNA, enriching mRNA was necessary by eliminating rRNA from the samples post-total RNA extraction using conventional kits. Oligo (dT) magnetic beads, followed by ultrasound-based blocking, achieved eukaryotic mRNA enrichment. The first cDNA strand was synthesized utilizing fragmented mRNA as a template and random oligonucleotides as primers within the M-MuLV reverse transcriptase system. Subsequently, the RNA strand was degraded using RNaseH, and the second cDNA strand was synthesized employing dNTPs in the DNA polymerase I system. The ru0bhaOAK2sulting double-stranded cDNA underwent purification, end-repair, A-tailing, and ligation to a sequence adapter. These constructed libraries were sequenced using Illumina HiSeqTM2000. These transcriptome data were assigned the accession number PRJNA1051837 and can be accessed in the NCBI GenBank database.

### 2.10. Statistical Analysis

All data were statistically validated using the one-way ANOVA with Tukey’s test for multiple comparisons. All statistical analyses were performed using GraphPad Prism 9.0. All data are expressed as the mean ± standard error (SEM), and *p* ≤ 0.05 indicates a significant difference.

## 3. Results

### 3.1. Growth Performance and Feed Utilization

The results of the WGR, SGR, PER, and FCR are listed in Table 2. The P44 group showed a significantly greater WGR and SGR than the P32 and P36 groups (*p* ≤ 0.05), which was the highest of all the groups. The FCR decreased with increasing protein levels in the P48 and P44 groups, demonstrating a significantly lower FCR than the P32 and P36 groups (*p* ≤ 0.05). The PER of the P44 and P48 groups were the highest among the groups (*p* ≤ 0.05), and no significant difference in the SR was observed among the different groups (*p* > 0.05).

### 3.2. Survival Rates of L. vannamei after WSSV and V. parahaemolyticus Infections

**Figure 1:**
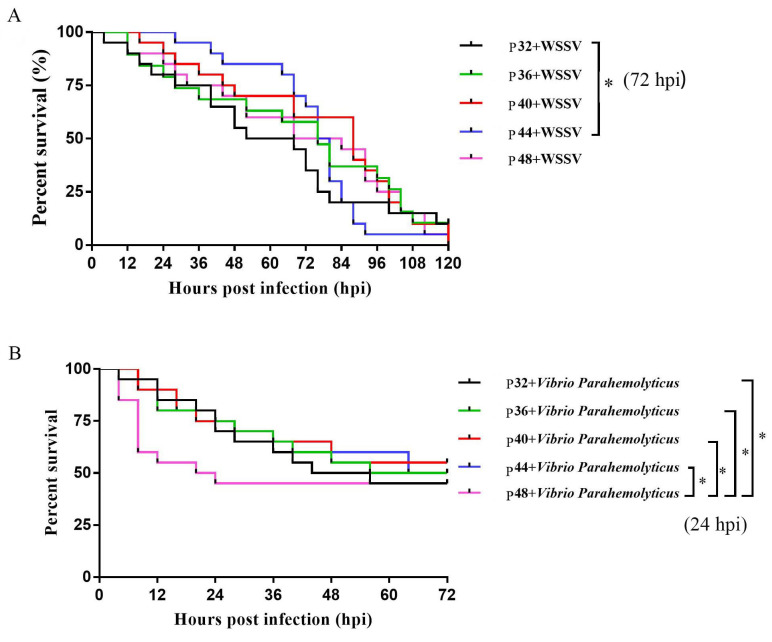
The survival rates of different protein levels fed *L. vannamei* after WSSV and *Vibrio parahaemolyticus* infections, (**A**) is the survival rate of *L. vannamei* at different protein levels after WSSV infection, (**B**) is the survival rate of *L. vannamei* at different protein levels after *Vibrio parahaemolyticus* infections. Differences in survival levels between treatments were analyzed using a Kaplan–Meier plot (log-rank χ^2^ test). Significant differences in the survival rate were marked with asterisks; * indicates *p* < 0.05.

The results of WSSV and *V. parahaemolyticus* infections are illustrated in Figure 1, which shows that the survival rate of the P44 group was significantly higher than that of the P32 group within 72 h of WSSV infection in shrimps (*p* ≤ 0.05). The survival rate of the P32, P36, P40, and P44 groups was significantly higher than that of the P48 group within 48 h of a *V. parahaemolyticus* infection (*p* ≤ 0.05).

### 3.3. Non-Specific Immune Indices

**Figure 2:**
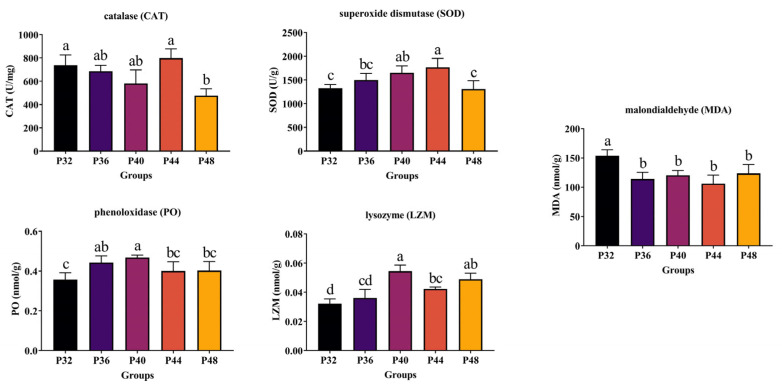
The effects of dietary protein levels on non-specific immune indices. Data were presented as the mean ± SD, *n* = 6. Values with different small letter superscripts mean significant differences between protein levels (*p* < 0.05).

The results in Figure 2 show that CAT levels in the P32 and P44 groups were significantly higher than in the P48 group (*p* ≤ 0.05). MDA was significantly higher in the P32 group than in the P36, P40, P44, and P48 groups (*p* ≤ 0.05). SOD in the P44 group was significantly higher than that in the P32 and P48 groups (*p* ≤ 0.05), and there were no significant differences between the P44 group and the P36 and P40 groups (*p* > 0.05). The PO level of the P40 group was significantly higher than that of the P32, P44, and P48 groups (*p* ≤ 0.05), with no significant differences observed between the P40 and P36 groups (*p* > 0.05). The LZM level of the P40 group was significantly higher than that of the P32, P36, and P44 groups (*p* ≤ 0.05), with no significant difference from the P48 group (*p* > 0.05).

### 3.4. Digestive Enzyme Activity

**Figure 3:**
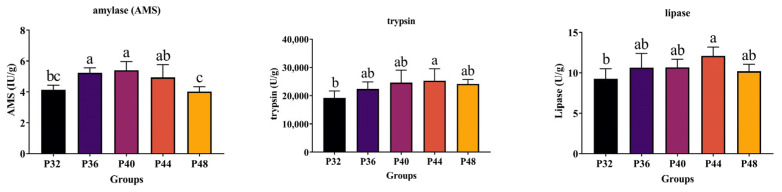
The effects of dietary protein levels on digestive enzyme activity. Data were presented as the mean ± SD, *n* = 6. Values with different small letter superscripts mean significant differences between protein levels (*p* < 0.05).

The data shown in Figure 3 demonstrate a positive correlation between protein levels and the increase in AMS, followed by a subsequent decrease. The values of the P36 and P40 groups were significantly higher than those of the P32 and P48 groups (*p* ≤ 0.05). However, there was no significant difference between the P36, P40, and P44 groups (*p* > 0.05). The levels of the P44 group were significantly higher in trypsin and lipase compared to the P32 group (*p* ≤ 0.05). Also, no significant differences were observed between the P44 group and the P36, P40, and P48 groups (*p* > 0.05).

### 3.5. Intestinal Microbiota Analysis

**Figure 4:**
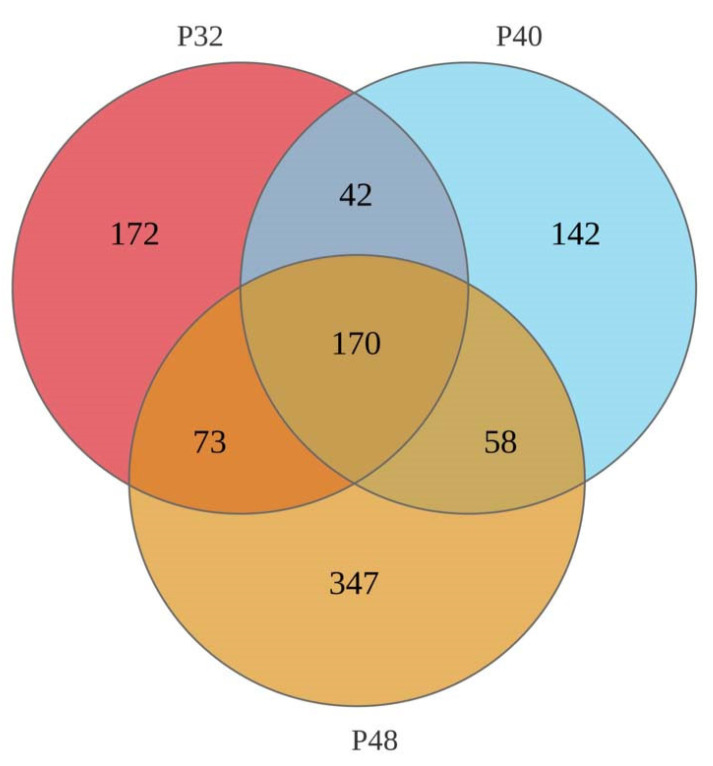
A Venn diagram of shared and unique OTUs of the intestinal microbiota in *L. vannamei*.

Based on the growth performance data of shrimp fed on diets with different protein levels, the low-protein group P32, the appropriate protein group P40, and the high-protein group P48 were selected for intestinal microbiota analysis. A Venn diagram was constructed to identify the differences and commonalities between these groups (Figure 4). A total of 170 operational taxonomic units (OTUs) were found in all the shrimp samples, while 172, 142, and 347 unique OTUs were observed in the P32, P40, and P48 groups, respectively. The P48 group contained the highest number of unique OTUs and the P40 group had the lowest number of OTUs. The effect of different protein levels in the feed on the alpha diversity of the intestinal microbiota of shrimp is shown in Table 3, with Good’s Coverage values above 99.80% in all groups. The Sobs, Chao, and Ace values for the P48 group were significantly higher than those for the P32 and P40 groups, and the Shannon index showed a significant difference between every group. In contrast, the Simpson index indicated no significant differences between them.

**Figure 5:**
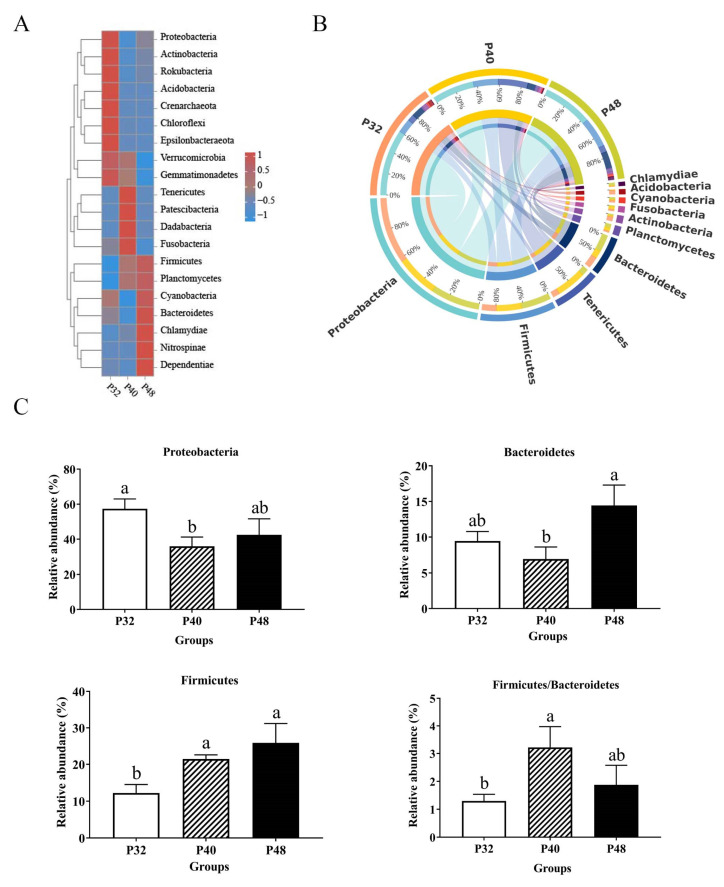
The effects of dietary protein levels on the intestinal microbiota of *L. vannamei* at the phylum level. (**A**) A heatmap of species abundance at the phylum level. Each row represents a species; each column represents a sample/grouping; and the colors represent species abundance. The closer the color is to dark blue, the lower the abundance. The closer the color is to red, the higher the abundance. The legend shows the corresponding species abundance values (or abundance values after normalizing the data). (**B**) A Circos graph of species at the phylum level. Grouping information is shown on one side of the graph and species information on the other. Lines on both sides indicate pairs of correspondences, with thicker lines indicating larger abundance values. (**C**) Relative abundance with significant differences in phylum levels. Values with different small letter superscripts mean significant differences between protein levels (*p* < 0.05).

**Figure 6:**
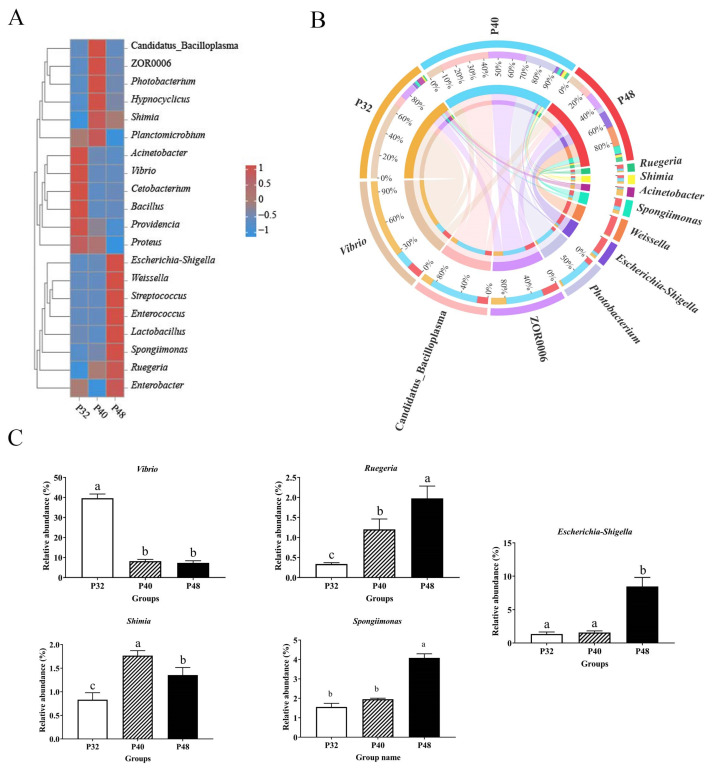
The effects of dietary protein levels on the intestinal microbiota of *L. vannamei* at the genus level. (**A**) A heatmap of species abundance at the genus level. (**B**) A Circos graph of species at the genus level. (**C**) Relative abundance with significant differences in genus levels. Values with different small letter superscripts mean significant differences between protein levels (*p* < 0.05).

At the phylum level (Figure 5), the dominant groups were Proteobacteria, Firmicutes, Tenericutes, Bacteroidetes, Planctomycetes, and Actinobacteria. The abundance of Proteobacteria was significantly lower in the P40 group than in the P32 group (*p* ≤ 0.05); Bacteroidetes was significantly lower than in the P48 group (*p* ≤ 0.05); and the values of Firmicutes and Firmicutes/Bacteroidetes were significantly higher than in the P32 group (*p* ≤ 0.05). At the genus level (Figure 6), *Vibrio*, Candidatus_Bacilloplasma, ZOR0006, *Photobacterium*, *Escherichia-Shigella*, *Weissella*, *Spongiimonas*, *Acinetobacter*, *Shimia*, and *Ruegeria* were the main dominant groups. Compared with the P32 group, the relative abundance levels of *Vibrio* and *Acinetobacter* in the other two groups were significantly decreased (*p* ≤ 0.05). However, the abundance levels of *spongiimonas* and *Escherichia-Shigella* in the P48 group were significantly higher than in the other two groups (*p* ≤ 0.05). It is worth mentioning that ZOR0006 and *Shimia* had the highest abundance in the P40 group.

### 3.6. Transcriptome Level Analysis

**Figure 7:**
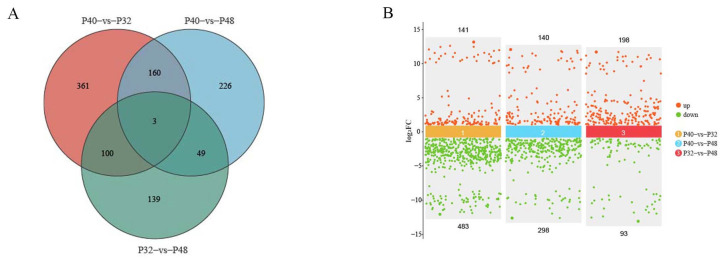
The effects of dietary protein levels on DEGs analysis. (**A**) A Venn diagram of the DEGs in *L. vannamei*. (**B**) A volcano diagram of the DEGs in different protein level-fed *L. vannamei*. The logarithmic value, log_2_(FC), of the multiple differences between groups is taken as the ordinate.

The results in Table 4 show that the mean values of raw reads obtained from transcriptome sequencing in the P32, P40, and P48 groups were 5,794,333,900, 5,653,803,200, and 5,777,191,300, respectively. After removing the reads with n ratios greater than 10% and low-quality primers, the mean net reads of 5,765,684,488, 5,622,266,515, and 5,746,684,868 were obtained. Comparing the tallied data with the reference genome yielded mean values of valid reads in the P32, P40, and P48 groups of 38,440,687, 37,484,161, and 38,313,659, respectively. The results shown in Figure 7 indicate that a total of 624 differentially expressed genes (DEGs) were identified between the P40 and P32 groups, with 141 up-regulated and 483 down-regulated genes. Similarly, DEGs were identified between the P40 and P48 groups, showcasing 140 up-regulated and 298 down-regulated genes. Lastly, 291 DEGs were identified between the P32 and P48 groups, featuring 198 up-regulated and 93 down-regulated genes. A two-by-two comparison between the three groups identified 361,226,139 DEGs unique to the P40 and P32 groups, P40 and P48 groups, and P32 and P48 groups, and 13 DEGs in common.

**Figure 8:**
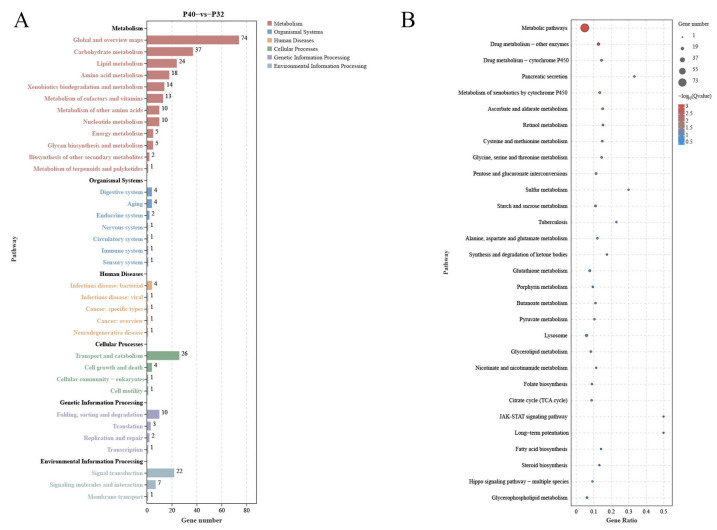
The analysis of the DEGs in the KEGG pathway in P40 vs. P32. (**A**) KEGG enrichment analysis of DEGs from P40 vs. P32. (**B**) Top 20 pathway significance bubble chart of P40 vs. P32; the bubble size indicates the number of differential genes enriched in the pathway, and the bubble color indicates the significance of enrichment in the pathway, with larger values indicating significant enrichment.

**Figure 9:**
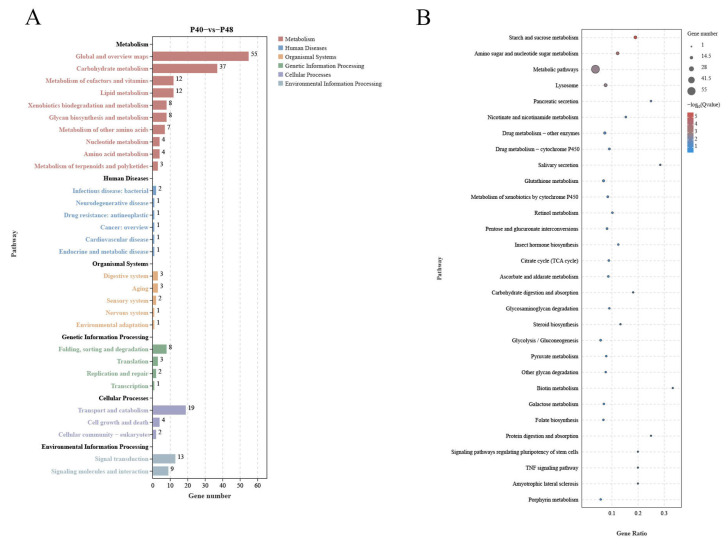
The analysis of the DEGs in the KEGG pathway in P40 vs. P48. (**A**) KEGG enrichment analysis of DEGs from P40 vs. P48. (**B**) Top 20 pathway significance bubble chart of P40 vs. P48.

The signaling pathways significantly enriched in the DEGs obtained from the two-by-two comparison of the experimental groups were classified into 40 subcategories using the KEGG database for annotation. Out of the 624 DEGs between the P40 and P32 groups (Figure 8), 213 were related to Metabolism, accounting for more than 34%, 14 to Organismal Systems (>4%), 8 to Human Disease (>1%), 32 to Cellular Processes (>5%), and 16 to Genetic Information Processing (>2%). In addition, 30 were related to Environmental Information Processing (>4%). Seventeen, one, zero, one, and one of the top twenty signaling pathways significantly enriched by KEGG were related to Metabolism, Cellular Processes, Environmental Information Processing, Organismal Systems, and Human Diseases, respectively. The top 5 signaling pathways were ko01100 (Metabolic Pathways), ko00983 (Drug Metabolism—Other Enzymes), ko00982 (Drug Metabolism—Cytochrome P450), ko04972 (Pancreatic Secretion), and ko00980 (Metabolism of Xenobiotics by Cytochrome P450). Out of the 438 DEGs between the P40 and P48 groups (Figure 9), 150 were related to Metabolism (>34%), 10 to Organismal Systems (>2%), 7 to Human Diseases (>1%), 25 to Cellular Processes (>5%), 14 to Genetic Information Processing (>3%), and 22 to Environmental Information Processing (with more than 5%). Sixteen, one, zero, three, and zero of the top 20 signaling pathways significantly enriched by KEGG were related to Metabolism, Cellular Processes, Environmental Information Processing, Organismal Systems, and Human Diseases, respectively. The top 5 signaling pathways were ko00500 (Starch and Sucrose Metabolism), ko00520 (Amino Sugar and Nucleotide Sugar Metabolism), ko01100 (Metabolic Pathways), ko04142 (Lysosome), and ko04972 (Pancreatic Secretion).

Several common pathways were present in the top 20 KEGG pathways for P40 vs. P32 and P40 vs. P48 (Figure 8 and Figure 9): Starch and Sucrose Metabolism, Nicotinate and Nicotinamide Metabolism, Metabolic Pathways, Drug Metabolism—Other Enzymes, Drug Metabolism—Cytochrome P450, Glutathione Metabolism, Retinol Metabolism, Pentose and Glucuronate Interconversions, Citrate Cycle (TCA cycle), Ascorbate and Aldarate Metabolism, Steroid Biosynthesis, Lysosome, and Pancreatic Secretion. The screening classification of the DEGs inside revealed 28 concurrent DEGs (Table 5) and pathways not directly related to nutrient metabolism: Drug Metabolism—Cytochrome P450, Glutathione Metabolism, Steroid Biosynthesis, Lysosome, and Pancreatic Secretion (details are added in Table 6).

## 4. Discussion

Growth performance is the most intuitive manifestation of an organism’s growth and development. Previous findings have investigated that *L. vannamei* requires, at least, a 32% protein level for optimal growth, whereas a 48% protein level in feed can create better feed efficiencies [33]. However, research by Gómez-Jiménez et al. and Xu et al. demonstrated no significant variations in the weight gain ratio (WGR) or digestibility of shrimp when protein levels in the feed ranged from 25% to 40% [11,34]. Given the considerable influence of environmental factors on nutritional requirements, the present study employed fish meal as the exclusive protein source to examine the effects of different protein levels on *L. vannamei*. Shrimp fed with a 44% protein content displayed the best growth performance compared to other experimental groups, with a higher PER, WGR, and SGR and a lower FCR. Notably, this study indicated that while increasing the feed protein level positively impacted growth performance to a certain extent, reaching 40% seemed to represent a threshold beyond which there was no substantial improvement in the digestive or immune performances of the shrimp. This finding aligns with that of Jang et al. [13].

Experiments were conducted under stress induced by *V. parahaemolyticus* and WSSV to assess the genuine impact of different protein levels on *L. vannamei*. The results showed that the P44 group was superior to the P32 group in terms of their WSSV stress response. Additionally, the P44 group showed a higher survival rate under *V. parahaemolyticus* infection than the P48 group. These findings further emphasized the importance of having the appropriate protein levels for enhancing immunity, with the shrimp being unable to maintain defense mechanism homeostasis in the face of a pathogenic infection when inappropriate protein levels were supplied.

Talukdar et al. noted an increase in trypsin activity in *L. vannamei* at higher dietary crude protein levels, which aligned with the present results [35]. In this experiment, differences in digestive enzymes were observed in response to the dietary absorption of different protein levels, with an increase in digestive enzyme activity observed when higher protein levels were ingested. Similarly, Xia et al. found that the protease and amylase activities of the shrimp intestines changed significantly when their dietary protein levels were increased, and that protease activity was positively correlated with dietary protein levels [36]. This was because as species grow, they consume more carbohydrates and lipids [37].

Shrimp possess a comprehensive antioxidative stress system that is crucial for maintaining homeostasis and amplifies the antioxidant response in adverse environments [38]. Inadequate protein levels can lead to malnutrition, sluggish growth, and increased vulnerability to bacterial disturbances. Conversely, excessive protein levels burden the hepatopancreas, inducing nutritional stress. Additionally, the release of surplus nutrients into the water can contribute to environmental pollution, and opportunistic pathogens within the body may damage vital organs such as the hepatopancreas [39,40]. LZM and PO are pivotal immune enzymes in the defense mechanism of *L. vannamei* [41,42]. In the present study, the activities of LZM and PO were most pronounced in the P40 group. Nutritional imbalances can result in oxidative damage, whereas optimal dietary protein levels mitigate the extent of such damage [5]. The content of MDA can often be indicative of the degree of lipid peroxidation and, by extension, the magnitude of cellular damage [43]. CAT and SOD, with their similar functions in eliminating excessive reactive oxygen species (ROS) from aquaculture systems, also play vital roles [44,45]. In the present study, the P32 group exhibited the highest hepatopancreatic MDA levels, while the activities of SOD and CAT were most pronounced in the P44 group. These findings suggest that optimal dietary protein levels can significantly reduce the susceptibility of *L. vannamei* to oxidative damage.

Utilizing 16S rDNA gene sequencing, the effects of three dietary protein levels (the P32, P40, and P48 groups) on the intestinal microbiome of *L. vannamei* were explored. It is generally accepted that high diversity in the intestine benefits the host’s health [46]. In this study, diets with different protein levels affected the abundance and diversity of intestinal microorganisms in the host, with inappropriate nutritional levels leading to an imbalance in the intestinal microbiota’s structure. Many previous studies have demonstrated that variations in dietary nutrient levels significantly affect the structure of the intestinal microbiota of shrimp. Liu et al. investigated the effects of two protein levels on silver pomfret larvae and observed significant effects on the structure of the intestinal microbiota [47]. Fan et al. also observed that the composition of the intestinal microbiota was significantly affected after using cottonseed protein to de-substitute different levels of fish meal as a dietary protein [48].

Firmicutes, Tenericutes, and Bacteroidetes are the most abundant phyla in prawns [49]. Studies have found that higher numbers of Firmicutes/Bacteroidetes are linked to superior growth performance and nutrient uptake in shrimp [50]. Proteobacteria are the largest bacterial phylum and the most extensively distributed in the marine environment, encompassing numerous harmful bacteria, and an increase in their abundance could lead to potential disease risks [51]. In this study, the abundance of Firmicutes in the P40 group was, the abundance of Tenericutes/Bacteroidetes was high, and the abundance of Proteobacteria phyla was the lowest, which may have been mainly due to the change in the dietary protein level.

At the genus level, the typical genus of pathogenic bacteria, *Vibrio*, contains several strains that can harm shrimp, such as *Vibrio harveyi* and *V. parahaemolyticus*. These can pose a significant threat to shrimp farming [52,53]. Additionally, *Acinetobacter* has been recognized as a conditional pathogen in *L. vannamei* [54]. In the present study, the abundance of *Acinetobacter* was significantly reduced when the protein level was increased, reducing the risk of disease in the shrimp. Furthermore, a significant boost in the abundance of two genera, *Ruegeria* and *Shimia*, was observed as the protein level increased. Most of the bacteria in these genera possess fundamental metabolic capabilities and partake in the host’s absorption and utilization of proteins [55]. Additionally, *Ruegeria* is antagonistic to some harmful bacteria and may be involved in the degradation of toxins and the production of beneficial metabolites [56]. Thus, the findings of the present study highlight the relationship between dietary protein levels and the intestinal microbiome, showcasing how alterations in the diet can significantly impact the abundance and diversity of microbial communities and potentially influence the health and disease susceptibility of *L. vannamei* shrimp.

Comparative transcriptome analyses can provide comprehensive insights into the systemic gene expression and regulation mechanisms of organisms. In the present study, the changes in host DEGs induced by changes in dietary protein levels revealed a significant increase in down-regulated DEGs relative to the P40 control at the two other protein levels, which exceeded the number of up-regulated DEGs. This observation suggested that inappropriate dietary protein levels may suppress the expression of specific genes, potentially resulting in imbalances between crucial biological functions. Ensuring a stable level of energy metabolism is paramount for an organism’s survival, with carbohydrate metabolism as a vital means of energy acquisition. The predominant pathways annotated by KEGG across the three treatment groups were the Metabolic Pathways, indicative of the notable impact of dietary protein levels on this function. By comparing the levels of differential DEGs present and using the data annotated in the KEGG database, many DEGs were found in response to changes in protein levels; some affected nutrient absorption by the organism. The 28 co-occurring DEGs observed in this study were most highly expressed in the P40 group, demonstrating that this group had a greater activity of more metabolic functions and was the most active of all the groups. Metabolic Pathways involve three significant nutrients: sugars, lipids, and proteins, while some pathways are not directly related to nutrient metabolism, such as Drug Metabolism—Other Enzymes, Drug Metabolism—Cytochrome P450, and Glutathione Metabolism.

Glutathione Metabolism is commonly associated with performance in response to environmental stressors, which plays a vital role in maintaining physiological homeostasis in shrimp. Glutathione (GSH) is an important antioxidant that helps to protect cells from damage caused by ROS and other harmful molecules [57]. In shrimp exposed to various environmental stressors, such as water quality, temperature, and salinity fluctuations, Glutathione Metabolism is essential for maintaining cellular health and protecting from oxidative stress [58,59]. The activity of this pathway in the P40 group and its relative inhibition in the P32 and P48 groups in the present study may be indicative of an enhancement in the antioxidant defense mechanism by the appropriate protein levels (P40 group). This meant that they could better neutralize ROS and other oxidative compounds, thereby reducing cellular damage and maintaining overall health. When Glutathione Metabolism in shrimp is optimal, shrimp may exhibit greater resistance to environmental stresses and diseases [60]. In contrast, the inhibition of the glutathione pathway may lead to an increased susceptibility to oxidative stress, reducing the ability of shrimp to detoxify harmful compounds and resist oxidative damage, which may lead to impaired health, reduced immune function, and increased susceptibility to diseases or environmental stressors. Thus, maintaining a balance in Glutathione Metabolism is essential for shrimp health and adaptation under varying ecological conditions. Factors affecting this pathway can significantly influence the ability of shrimp to cope with stress and maintain overall health.

A pathway that is associated with immune factors, the Drug Metabolism—Cytochrome P450 pathway, is representative of the intricate biochemical processes that are responsible for the metabolism of a diverse array of compounds wherein cytochrome P450 enzymes play a pivotal role [61]. Studies have shown that under environmental stressors, such as temperature fluctuations or exposure to pathogens, the expression and activity of cytochrome P450 enzymes may undergo alterations in organisms [62,63]. When the functionality of these enzymes is impaired, organisms may become more susceptible to environmental pollutants, rendering them more prone to the detrimental effects of pollution [64]. In the present study, multiple DEGs within this pathway were observed to be down-regulated in both the P32 and P48 groups. This impairment in the in vivo homeostasis of this pathway could render organisms more susceptible to foreign pathogens. This may be one of the contributing factors to the lower survival rate observed in the P32 and P48 groups during the challenge tests, as well as the higher abundance of harmful bacteria in the intestinal environment.

Two extra Metabolic Pathways are worth noting: Lysosome and Pancreatic Secretion. The Lysosome pathway governs the acceptance and catabolism of macromolecules from the secretory, autophagic, and endocytic membrane transport pathways [65,66]. These processes are intricately involved in regulating various biological functions, including energy metabolism and cellular homeostasis. In the P32 and P48 groups, multiple DEGs related to this pathway were also significantly down-regulated. While invertebrates like shrimp lack a pancreas akin that of mammals, they possess a specialized digestive structure known as the hepatopancreas. Functionally, this organ is the liver and the pancreas, responsible for producing and secreting digestive enzymes into the stomach to facilitate food breakdown [67]. Although the Pancreatic Secretion pathway defined in KEGG may not directly apply to invertebrates, particularly shrimp, KEGG annotations for these organisms emphasize specific physiological and biochemical processes that are unique to them. Notably, Jiang et al. observed that an elevated content of *Clostridium autoethanogenum* protein adversely affected *L. vannamei* by influencing pancreatic secretion [68]. In our investigation, within the pancreatic secretory pathway, suppressed expression levels of *amy*, *cpa1*, and *cla2* in the P32 group and *amy* and *cpa*1 in the P48 group were observed compared to the P40 group. The genes *amy* and *cpa1* are integral to the secretion of amylase and protease, respectively [69,70]. *Cap1* encodes a member of the zinc metalloproteinase family of carboxypeptidases and is primarily synthesized in the pancreas, specializing in breaking C-terminal-branched chains and aromatic amino acids in dietary proteins [71]. *Amy* is pivotal in starch and sucrose metabolism, metabolic pathways, salivary secretion, and pancreatic secretion, as well as the carbohydrate digestion and absorption pathways [72]. Both enzymes are crucial for nutrient digestion and absorption, which may directly affect protein and starch absorption. Their down-regulation could trigger a series of malnutritional reactions, ultimately impacting the digestive function of the shrimp hepatopancreas; the observed reduction in digestive enzyme activity in the P32 and P48 groups may be attributed to this underlying mechanism. In the P32 group, inhibition of the gene *clca2* was observed, which also implicated the involvement of the Renin secretion pathway in vivo. *Clca2* encodes a member of the calcium-activated chloride channel regulatory protein (CLCR) family, known for its role in regulating the transport of chloride ions across the plasma membrane [73,74]. Additionally, it has been noted that *clca2* can moderately stimulate intracellular calcium pool release [75]. However, the specific mechanisms behind this phenomenon remain to be elucidated. In summary, within the experimental framework of this study, which encompassed three different protein levels, it was observed that inappropriate protein levels may detrimentally impact *L. vannamei* by disrupting its immune homeostasis.

## 5. Conclusions

In this study, the diet containing around 44% protein resulted in superior growth performance, disease resistance, digestion, and immunity of *L. vannamei* compared to the other diets. Changes in the intestinal microbiota showed an increase in some harmful bacteria and a decrease in beneficial bacteria when the feed contained only 32% protein. Transcriptomic analysis revealed that the DEGs in groups with different protein levels were mainly associated with metabolism and development, as indicated by the KEGG enrichment analysis. The results of this study may provide theoretical guidance for the precise nutrition of *L. vannamei*.

## Figures and Tables

**Table 1:** The formula and proximate composition of the basal diet (dry matter basis, g/kg).

Ingredient (g/kg)	Groups				
	P32	P36	P40	P44	P48
Fishmeal	471	530	589	647.5	706
Corn starch	150	150	150	150	150
Fish oil	9.5	6.55	3.6	0.68	0
Corn oil	9.5	6.55	3.6	0.68	0
Soyabean lecithin	10	10	10	10	5.5
Vitamin and mineral premix ^a^	12	12	12	12	12
Choline chloride	5	5	5	5	5
Antioxidant ^b^	0.5	0.5	0.5	0.5	0.5
Attractant ^c^	1	1	1	1	1
CaH_2_PO_4_·H_2_O	12	12	12	12	12
Vitamin C	0.5	0.5	0.5	0.5	0.5
Cellulose	319	265.9	212.8	160.14	107.5
Total	1000.0	1000.0	1000.0	1000.0	1000.0
**Nutrient index ^d^**	**Proximate composition (%)**				
Crude protein	33.02	37.27	40.73	45.94	47.76
Crude lipid	6.15	6.41	6.69	6.87	7.56
Crude ash	8.4	9.2	10.4	11.6	12.7
Moisture	9	9	9	9	9

Note: ^a^ The vitamin and mineral premix (kg^−1^ of diet) includes the following contents: thiamine, 5 mg; riboflavin, 10 mg; vitamin A, 5000 IU; vitamin D3, 1000 IU; vitamin E, 40 mg; menadione, 10 mg; pyridoxine, 10 mg; biotin, 0.1 mg; cyanocobalamin, 0.02 mg; calcium pantothenate, 20 mg; folic acid, 1 mg; niacin, 40 mg; vitamin C, 150 mg; FeSO_4_·H_2_O, 303 mg; KIO_3_, 1.3 mg; Cu_2_(OH)_3_Cl, 5 mg; ZnSO_4_·H_2_O, 138 mg; MnSO_4_·H_2_O, 36 mg; Na_2_SeO_3_, 0.6 mg; and CoCl_2_·6H_2_O, 0.8 mg; ^b^ The antioxidant is ethoxyquinoline (EQ) (%), 52.0–60.0; propyl gallate vinegar (PG) (%), 2.0–5.0%. ^c^ The attractant is betaine; ^d^ the nutrient index is the average of the actual test values.

**Table 2:** Effects of different treatment groups on the growth performance of *L. vannamei*.

Item	Groups				
	P32	P36	P40	P44	P48
IBW (g)	0.63 ± 0.02	0.63 ± 0.02	0.63 ± 0.02	0.63 ± 0.02	0.63 ± 0.02
FBW (g)	6.58 ± 0.30	7.03 ± 0.36	7.6 ± 0.75	8.44 ± 0.16	7.89 ± 0.07
WGR (%)	934.81 ± 47.00 ^c^	1011.25 ± 56.86 ^bc^	1095.83 ± 116.90 ^abc^	1231.5 ± 30.81 ^a^	1142.76 ± 8.05 ^ab^
SR (%)	91.67 ± 1.44	96.67 ± 1.44	93.33 ± 6.29	94.17 ± 5.20	94.17 ± 5.77
SGR (%)	4.17 ± 0.08 ^c^	4.3 ± 0.09 ^bc^	4.43 ± 0.18 ^abc^	4.62 ± 0.04 ^a^	4.5 ± 0.01 ^ab^
FCR	4.42 ± 0.32 ^a^	3.94 ± 0.20 ^ab^	3.42 ± 0.31 ^bc^	3.06 ± 0.15 ^c^	2.99 ± 0.17 ^c^
PER (%)	7.27 ± 0.52 ^c^	9.15 ± 0.48 ^c^	11.76 ± 1.07 ^b^	14.4 ± 0.68 ^a^	16.09 ± 0.92 ^a^

Note: Data were presented as the mean ± SD, *n* = 3. No superscript or the same superscript in the same line means no significant difference (*p* > 0.05). Values with different superscripts in the same row differ significantly (*p* < 0.05). IBW: initial average weight. FBW: final average weight. WGR: specific growth rate. SR: specific growth rate. SGR: specific growth rate. FCR: feed conversion ratio. PER: protein efficiency ratio.

**Table 3:** Alpha diversity-related indexes of *L. vannamei*.

Index	Groups		
	P32	P40	P48
Sobs	423.33 ± 20.55 ^b^	383 ± 23.64 ^b^	507.67 ± 29.02 ^a^
Shannon	3.08 ± 0.09 ^c^	3.64 ± 0.09 ^b^	3.92 ± 0.06 ^a^
Simpson	0.8 ± 0.03	0.78 ± 0.05	0.87 ± 0.04
Chao	657.87 ± 35.72 ^b^	659.51 ± 33.75 ^b^	747.61 ± 23.67 ^a^
Ace	741.15 ± 21.33 ^b^	730.47 ± 16.39 ^b^	792.87 ± 19.63 ^a^

Notes: Different small letter superscripts mean a significant difference (*p* < 0.05); no letter labeling indicates no significant difference (*p* > 0.05).

**Table 4:** Statistics of transcriptome sequencing in *L. vannamei*.

Sample	All Reads-Raw Data (bp)	Q20 (%)	Q30 (%)	GC (%)	Filter-Clean Data (%)
P32-1	6,111,628,800	97.88%	93.75%	43.42%	40,568,650 (99.57%)
P32-2	5,780,577,000	97.86%	93.70%	44.59%	38,366,864 (99.56%)
P32-3	5,490,795,900	97.75%	93.53%	44.25%	36,386,546 (99.40%)
P40-1	5,771,514,600	97.98%	94.07%	43.26%	38,295,220 (99.53%)
P40-2	6,010,638,000	98.02%	94.13%	44.31%	39,829,030 (99.40%)
P40-3	5,179,257,000	97.57%	93.13%	45.92%	34,328,232 (99.42%)
P48-1	5,367,786,300	98.00%	94.11%	43.48%	35,587,522 (99.45%)
P48-2	5,600,367,300	97.92%	93.97%	45.02%	37,140,840 (99.48%)
P48-3	6,363,420,300	98.05%	94.16%	42.75%	42,212,616 (99.50%)

**Table 5:** Common DEGs in the top 20 pathways co-annotated to P40 vs. P32 and P40 vs. P48.

DEGs	Description	P32 Mean (fpkm)	P40 Mean (fpkm)	P48 Mean (fpkm)
ROT61949.1	amy	0.17	1.00	0.03
ROT75446.1	*enpp3*	3.62	8.00	1.51
MSTRG.31007	*amy*	0.33	3.89	0.33
MSTRG.31768	*celd*	0.50	4.92	0.90
ROT62696.1	*rrm1*	2.80	24.23	5.62
ROT66027.1	*pck2*	9.77	27.33	6.42
ROT67232.1	*pnlip*	1.33	6.61	1.45
ROT67236.1	*inpp4a*	11.10	5.32	12.72
ROT68435.1	*tpi1a*	4.21	0.00	2.80
ROT68492.1	*rgn*	1.84	8.90	3.73
ROT71552.1	*acss3*	0.22	2.25	0.18
ROT72812.1	*smpd1*	0.08	1.47	0.24
ROT73315.1	*ugt2b16*	2.15	6.91	2.50
ROT76522.1	*chia*	1.18	6.07	1.53
ROT77738.1	*sam-s*	5.31	78.44	26.02
ROT77960.1	*amy1*	0.96	6.31	0.97
ROT79971.1	*ugt8*	1.93	5.92	1.56
ROT80223.1	*akr1b1*	5.85	21.78	6.80
ROT81932.1	*--*	0.86	4.75	1.27
ROT82637.1	*pnliprp2*	0.11	2.55	0.33
MSTRG.26644	*hexb*	5.71	13.32	6.04
MSTRG.30076	*rrm1*	1.26	10.50	2.78
MSTRG.4293	*scsalpha1*	1.68	0.05	3.20
ROT79533.1	*gstd1*	2.83	14.90	4.57
ROT61670.1	*gpx*	0.66	7.74	0.94
MSTRG.22898	*se*	6.28	14.56	6.88
ROT70506.1	*lip3*	0.75	4.92	0.74
ROT80984.1	*lipf*	20.37	57.51	14.35

Notes: The mean (fpkm) is the mean fragments per kilobase of exon model per million mapped fragments within this group.

**Table 6:** The effects of different treatment groups on the KEGG pathways (Lysosome, Pancreatic Secretion, and Drug Metabolism—Cytochrome P450) of *L. vannamei*.

Gene ID	log2(fc)	Symbol	Description
**Glutathione Metabolism**			
**P40 vs. P32**			
ROT61670.1	−3.54	*gpx*	Glutathione peroxidase 3 (*Penaeus monodon*)
ROT62696.1	−3.11	*rrm1*	PREDICTED: ribonucleoside-diphosphate reductase large subunit-like (*Hyalella azteca*)
ROT79532.1	−1.25	*gstd1*	Delta-class glutathione S-transferase (*Fenneropenaeus chinensis*)
ROT79533.1	−2.40	*gstd1*	Delta-class glutathione S-transferase (*Fenneropenaeus chinensis*)
ROT81832.1	0.87	*gclc*	PREDICTED: glutamate--cysteine ligase catalytic subunit-like (*Hyalella azteca*)
MSTRG.22898	−1.21	*se*	Pyrimidodiazepine synthase-like (*Penaeus vannamei*)
MSTRG.30076	−3.06	*rrm1*	Ribonucleoside diphosphate reductase large subunit-like (*Penaeus vannamei*)
**P40 vs. P48**			
ROT61670.1	−3.04	*gpx*	Glutathione peroxidase 3 (*Penaeus monodon*)
ROT62696.1	−2.11	*rrm1*	PREDICTED: ribonucleoside diphosphate reductase large subunit-like (*Hyalella azteca*)
ROT71141.1	−1.95	*gstd1*	Delta-class glutathione S-transferase (*Fenneropenaeus chinensis*)
ROT72062.1	−0.48	*anpep*	PREDICTED: aminopeptidase N-like (*Hyalella azteca*)
ROT78750.1	−1.07	*gstm1*	Glutathione S-transferase (*Litopenaeus vannamei*)
ROT79533.1	−1.71	*gstd1*	Delta-class glutathione S-transferase (*Fenneropenaeus chinensis*)
MSTRG.22898	−1.08	*se*	Pyrimidodiazepine synthase-like (*Penaeus vannamei*)
MSTRG.30076	−1.92	*rrm1*	Ribonucleoside diphosphate reductase large subunit-like (*Penaeus vannamei*)
**Drug Metabolism—Cytochrome P450**			
**P40 vs. P32**			
ROT71141.1	−3.74	*gstd1*	Delta-class glutathione S-transferase (*Fenneropenaeus chinensis*)
ROT72759.1	−4.29	*ugt2b13*	PREDICTED: UDP-glucuronosyltransferase 2B14-like (*Hyalella azteca*)
ROT73315.1	−1.68	*ugt2b16*	PREDICTED: UDP-glucuronosyltransferase-like isoform X2 (*Hyalella azteca)*
ROT74229.1	−4.52	*ugt8*	PREDICTED: 2-hydroxyacylsphingosine 1-beta-galactosyltransferase-like (*Hyalella azteca*)
ROT78750.1	−2.52	*gstm1*	Glutathione S-transferase (*Litopenaeus vannamei*)
ROT79533.1	−2.40	*gstd1*	Delta-class glutathione S-transferase (*Fenneropenaeus chinensis*)
ROT79971.1	−1.62	*ugt8*	PREDICTED: UDP-glucuronosyltransferase 2B19-like isoform X1 (*Hyalella azteca*)
ROT80959.1	−3.98	*ugt1a8*	PREDICTED: UDP-glucuronosyltransferase 2B19-like isoform X1 (*Hyalella azteca*)
**P40 vs. P48**			
ROT73315.1	−1.47	*ugt2b16*	PREDICTED: UDP-glucuronosyltransferase-like isoform X2 (*Hyalella azteca*)
ROT74228.1	−3.40	*ugt8*	PREDICTED: 2-hydroxyacylsphingosine 1-beta-galactosyltransferase-like (*Hyalella azteca*)
ROT79532.1	−2.99	*gstd1*	Delta-class glutathione S-transferase (*Fenneropenaeus chinensis*)
ROT79533.1	−1.71	*gstd1*	Delta-class glutathione S-transferase (*Fenneropenaeus chinensis*)
ROT79971.1	−1.92	*ugt8*	PREDICTED: UDP-glucuronosyltransferase 2B19-like isoform X1 (*Hyalella azteca*)
**Lysosome**			
**P40 vs. P32**			
ROT62942.1	−2.46	*ctsc*	Cathepsin C (*Fenneropenaeus chinensis*)
ROT64929.1	−9.56	*asm-2*	PREDICTED: sphingomyelin phosphodiesterase-like (*Hyalella azteca*)
ROT70506.1	−2.72	*lip3*	Triacylglycerol lipase (*Litopenaeus vannamei*)
ROT72812.1	−4.26	*smpd1*	PREDICTED: sphingomyelin phosphodiesterase-like (*Hyalella azteca*)
ROT73988.1	−9.94	*lcp2*	Cathepsin L (*Marsupenaeus japonicus*)
ROT79070.1	−2.90	*ctsc*	Cathepsin C (*Fenneropenaeus chinensis*)
ROT80984.1	−1.50	*lipf*	Triacylglycerol lipase (*Portunus trituberculatus*)
ROT83980.1	−2.28	*arsa*	PREDICTED: arylsulfatase A-like (*Hyalella azteca*)
ROT85091.1	−1.99	*man2b1*	PREDICTED: lysosomal alpha-mannosidase-like (*Hyalella azteca*)
ROT85637.1	−4.11	*npc1*	PREDICTED: Niemann–Pick C1 protein-like (*Hyalella azteca*)
MSTRG.26644	−1.22	*hexb*	Beta-hexosaminidase subunit alpha-like (*Penaeus vannamei*)
**P40 vs. P48**			
ROT61198.1	−2.96	*aael006169*	Cathepsin D-like protein (*Homarus americanus*)
ROT62149.1	−1.97	*brafldraft_56888*	PREDICTED: alpha-L-fucosidase-like (*Hyalella azteca*)
ROT62942.1	−2.61	*ctsc*	Cathepsin C (*Fenneropenaeus chinensis*)
ROT66187.1	−4.72	*arsb*	PREDICTED: arylsulfatase B-like (*Branchiostoma belcheri*)
ROT67034.1	−2.55	*arsb*	PREDICTED: arylsulfatase B-like (*Branchiostoma belcheri*)
ROT70506.1	−2.73	*lip3*	Triacylglycerol lipase (*Litopenaeus vannamei*)
ROT70922.1	−2.51	*smpd1*	PREDICTED: sphingomyelin phosphodiesterase-like (*Diachasma alloeum*)
ROT72812.1	−2.62	*smpd1*	PREDICTED: sphingomyelin phosphodiesterase-like (*Hyalella azteca*)
ROT73985.1	−3.71	*lcp2*	Cathepsin l, partial (*Litopenaeus vannamei*)
ROT73986.1	−2.78	*lcp2*	Cathepsin l (*Litopenaeus vannamei*)
ROT80984.1	−2.00	*lipf*	Triacylglycerol lipase (*Portunus trituberculatus*)
ROT84188.1	−9.15	*slc17a2*	PREDICTED: sialin-like (*Hyalella azteca*)
ROT85091.1	−2.40	*man2b1*	PREDICTED: lysosomal alpha-mannosidase-like (*Hyalella azteca*)
MSTRG.26644	−1.14	*hexb*	Beta-hexosaminidase subunit alpha-like (*Penaeus vannamei*)
**Pancreatic Secretion**			
**P40 vs. P32**			
MSTRG.26333	−5.19	--	Group 3 secretory phospholipase A2-like (*Penaeus vannamei*)
MSTRG.31007	−3.56	*amy*	Amylase (*Penaeus vannamei*)
MSTRG.31195	−3.54	*cpa1*	Carboxypeptidase B-like (*Penaeus vannamei*)
MSTRG.31546	−1.77	*clca2*	Calcium-activated chloride channel regulator 4A-like isoform X2 (*Penaeus vannamei*)
**P40 vs. P48**			
MSTRG.31001	−3.15	*amy*	LOW-QUALITY PROTEIN: alpha-amylase-like (*Penaeus vannamei*)
MSTRG.31007	−3.57	*amy*	Amylase (*Penaeus vannamei*)
MSTRG.31195	−2.00	*cpa1*	Carboxypeptidase B-like (*Penaeus vannamei*)

## Data Availability

The data that support the findings of this study are available on request from the corresponding author. The data are not publicly available due to privacy or ethical restrictions.
